# Use of bmps as a treatment for medication-related maxillary osteonecrosis (mronj): a systematic review

**DOI:** 10.2340/aos.v85.45323

**Published:** 2026-02-04

**Authors:** Lucía Hernando-Calzado, Aida Bauer-González, Carlos Manuel Cobo-Vázquez, Cristina Meniz-García, Juan López-Quiles, Cristina Madrigal Martínez-Pereda

**Affiliations:** a Department of Dental Clinical Specialties, Faculty of Dentistry, Complutense University of Madrid, Madrid, Spain; b Surgical and Implant Therapies in the Oral Cavity Research Group, University Complutense, Madrid, Spain

**Keywords:** MRONJ, medication-related osteonecrosis, bone regeneration, BMPs, bone morphogenetic protein

## Abstract

**Background:**

Medication-related osteonecrosis of the jaw (MRONJ) is an adverse condition in patients receiving antiresorptive or antiangiogenic therapies. Standard treatments, including surgical debridement, often yield suboptimal outcomes. In this context, bone morphogenetic proteins (BMPs), have been explored for their ability to stimulate osteogenesis and enhance bone repair.

**Materials and methods:**

A systematic review of the literature was conducted, focusing on studies that applied rh-BMPs during surgeries to treat MRONJ. Databases were searched for relevant articles from inception to the present, using keywords such as ‘MRONJ’, ‘BMP’, and ‘bone regeneration’. Inclusion criteria involved studies with human participants who had been treated with rh-BMPs, along with the surgical elimination of bone sequestrum, MRONJ stages 2 and 3 according to the AAOMS staging system and a minimum follow-up period of 6 months. Two independent reviewers (L.H.C. and C.C.V.) systematically selected the articles independently.

**Results:**

The review included nine studies with a total of 217 patients treated with rh-BMP. Bone regeneration and osteonecrosis healing was reported in all the studies included using rh-BMP. However, the measurement methods were very different between the studies, using clinical examinations, different radiological tests and biomarkers and own scales. Moreover, there were inconsistencies in treatment protocols and follow-up periods, making it difficult to standardize conclusions.

**Discussion:**

While rh-BMPs show promising results for bone regeneration in MRONJ patients, the variability in study methodologies limits definitive conclusions. The biological potential of BMPs could be beneficial, but standardized protocols and longer-term studies are needed to establish their effectiveness.

**Conclusions:**

The application of rh-BMPs may promote bone regeneration in MRONJ patients, but further research with standardized methods is required to confirm these findings.

## Introduction

Since their discovery in 1969, bisphosphonates have become widely used medications for the treatment of conditions such as osteoporosis and bone metastases [1]. However, adverse effects such as osteonecrosis of the jaw have been reported. In 2003, Robert Marx first described this condition, naming it bisphosphonate-related osteonecrosis of the jaw (BRONJ) [2–5]. The American Association of Oral and Maxillofacial Surgeons (AAOMS) later revised the terminology to medication-related osteonecrosis of the jaw (MRONJ) to include cases linked to other antiresorptive and antiangiogenic agents. Diagnostic criteria for MRONJ include current or past exposure to these drugs, bone exposure or a probing fistula in the maxillofacial region persisting for more than 8 weeks, and the exclusion of prior radiation therapy or jaw metastasis [2].

Epidemiologically, MRONJ has a higher incidence in males, with a sex ratio of 1.2:1 [3]. Reported prevalence ranges from 0.3% to 6.7%, influenced by the type and duration of treatment [2, 4–6]. Systematic reviews indicate incidence rates of 0.9% to 3.1% among patients receiving bisphosphonates or denosumab [7–10]. Clinically, MRONJ is classified into four stages (0–3). Stage 0 shows no exposed bone but presents clinical or radiographic abnormalities. Stage 1 involves exposed bone without symptoms. Stage 2 is marked by exposed necrotic bone with pain and infection. Stage 3 includes extensive necrosis beyond the alveolar bone [2].

Management remains stage-specific and lacks a universal consensus. The AAOMS recommends conservative therapy (e.g. chlorhexidine rinses, systemic antibiotics) for stages 0 and 1. In stage 2, surgical debridement may complement conservative management. For stage 3, more aggressive surgical interventions, including bone resection, are indicated to control infection and pain [2, 11].

Such interventions often result in significant bone defects, prompting the need for reconstructive techniques. Autogenous bone grafts remain the gold standard due to their osteoconductive and osteoinductive properties, but they carry risks such as donor site morbidity and limited tissue availability [12]. This has led to growing interest in growth factor-based therapies, including autologous platelet concentrates (APC) and recombinant proteins such as platelet-derived growth factor (PDGF) and bone morphogenetic proteins (BMPs) [13].

BMPs, members of the transforming growth factor-β (TGF-β) family, induce mesenchymal stem cell differentiation into osteoblasts [12]. Among them, BMP-2, -4, -6, -7, and -9 are most relevant in bone regeneration [14, 15]. Both clinical and preclinical studies suggest that recombinant human BMP-2 (rhBMP-2) is a promising and safe alternative for enhancing bone regeneration in MRONJ patients undergoing surgical treatment [16–18].

Although case series and case reports in humans exist, most of the literature is based on preclinical animal studies. No systematic review has been conducted to our knowledge that comprehensively evaluates the use of rh-BMP2 in bone regeneration in patients with MRONJ. This article is the first systematic review dedicated to analyzing the available evidence on this topic to provide a clearer evaluation of its clinical applicability.

The objective of this systematic review is to study the outcomes in terms of bone regeneration following the application of rh-BMPs in patients undergoing surgeries for the removal of bone sequestrum caused by MRONJ.

## Material and methods

### Protocol development and PICO question

This study was conducted in accordance with the PRISMA statement (Preferred Reporting Items for Systematic Reviews and Meta-Analysis) and is registered in the International Prospective Register of Systematic Reviews (PROSPERO) (CRD42024571334) [19].

The PICO question that guided the study objective was: in patients undergoing sequestrectomy surgeries for maxillary osteonecrosis (P), are there differences in terms of bone regeneration (O) following the application of BMPs (I) compared to conventional surgical treatment (C)?

### Selection criteria

#### Inclusion criteria

- Clinical studies in humans
- Randomized clinical trials, cohort studies, case reports, case series, observational studies, case-control studies
- MRONJ stages 2 and 3 according to the AAOMS staging system
- Minimum follow-up period of 6 months
- Articles published in English or Spanish up to July 1, 2024

#### Exclusion Criteria

- Animal studies
- In vitro studies
- Review articles
- Osteonecrosis located outside the maxilla and mandible

### Search strategy

Electronic databases PubMed (MEDLINE), Scopus (Elsevier), The Cochrane Library (Wiley), and Web of Science (Clarivate Analytics) were searched for articles published in English or Spanish up to July 1, 2024, without publication date restrictions. The search strategy for PubMed was a combination of MeSH terms and keywords in advanced mode without filters: ((bone morphogenetic protein) OR (BMP) OR (BMPs)) AND ((MRONJ) OR (drug-related osteonecrosis) OR (bisphosphonates osteonecrosis)). The search strategy was adapted for each database. The search was supplemented by manually reviewing the references cited in the selected articles and in similar review articles (Appendix 1).

### Study selection

Two independent reviewers (L.H.C. and C.C.V.) systematically selected the articles independently. The process began with a title review, followed by an abstract review, and finally, the full-text articles were evaluated to determine if they met the inclusion criteria. In case of disagreement, a third researcher (J.L.Q.) was consulted to decide on the inclusion or exclusion of articles. Zotero (Center for History and New Media, George Mason University, Virginia, USA) was used to detect duplicate references. Agreement between reviewers on study inclusion was assessed using the Kappa index, with agreement defined as slight (0.00 to 0.20), fair (0.21 to 0.40), moderate (0.41 to 0.60), good (0.61 to 0.80), or excellent (0.81 to 1.00).

### Data extraction and analysis method

Qualitative and quantitative data from all included studies were independently extracted using data extraction tables in Microsoft Excel^®^ 2019 (Microsoft Corporation, Redmond, Washington, USA). The qualitative variables recorded were: author, study design, most commonly used antiresorptive medication, reason for treatment, BMP brand name, types of tests and measurements performed on patients. The quantitative variables collected were: year, total number of patients, number of patients treated with BMP, BMP dose administered, radiographic index and its variation, bone regeneration rate, corrected bone regeneration rate, maximum clinical follow-up, maximum radiographic follow-up with panoramic views and Cone Beam Computed Tomography (CBCT). In cases where information was unavailable, the corresponding authors were contacted. If no response was received, the variable was considered NA (not available).

### Study quality assessment

The quality of the studies was analyzed. For cohort and case-control studies, the Newcastle-Ottawa Scale (NOS) was used [20]. This scale includes three main categories: selection of study groups, comparability between participants, and exposure to the variable under study. Each study can receive a maximum of nine points. For case series and case reports, the Joanna Briggs Institute (JBI) Critical Appraisal Checklist was used, consisting of 10 or eight questions that assess the methodological aspects of the article, as well as the reporting of results [21]. Lastly, the quality of Randomized Controlled Trial’s (RCT) was examined using the Jadad scale, which consists of five yes-or-no questions, yielding a score between 0 and 5 [22].

## Results

### Study selection

**Figure 1:**
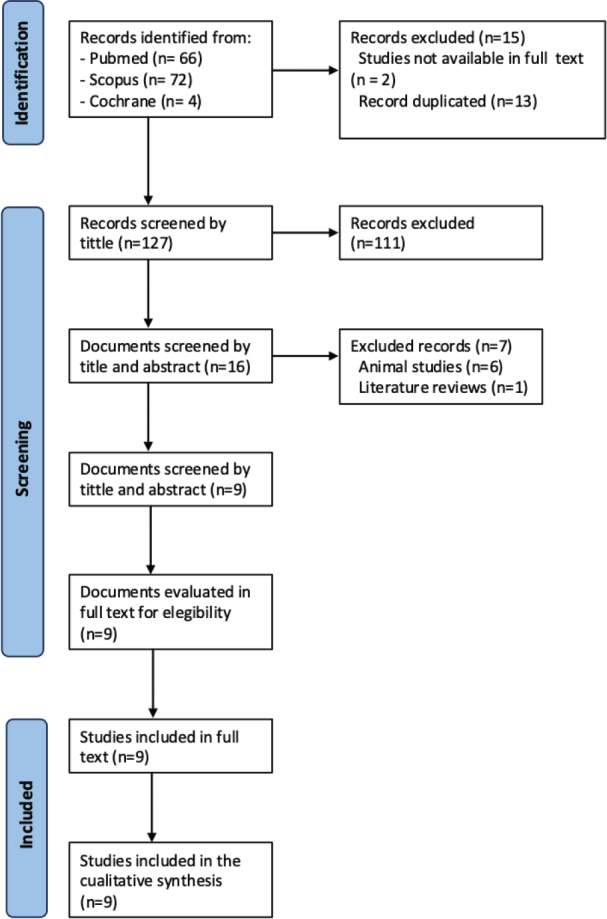
Flow chart.

A total of 142 articles were identified in the electronic search after applying the established filters. After duplicates were removed, 127 articles were screened by title. After applying the inclusion and exclusion criteria, a total of nine articles were reviewed. The search and selection process is represented in the PRISMA flow diagram (Figure 1). The Kappa index was calculated, showing an excellent level of agreement between the authors (κ = 1.0).

### Study’s characteristics

Nine full-text articles were included in this systematic review: one RCTs [18], four case reports [17, 23–25], two retrospective studies [26, 27], one case series [16] and one prospective study [28]. The selected studies were published between May 2012 and July 2024. Table 1 describes the main characteristics of the selected studies.

**Table 1:** Description of the selected studies.

Author	Year	Study desing	Total patients	Patients BMP alone	Patients BMP + X	Type of antiresorptive	Reasson for biphosphonate use	Dosage BMP	Tests performed	Evaluation regeneration	Maximux follow up period
Kim et al.	2024	Case control	93	25	-	Alendronate (36.6%)	Osteoporosis (100%)	1.5 mg rhBMP-2	Clinical review + panoramic X-Ray + CBCT	Own scale	6 months
Moraes Da Silva et al.	2022	Case report	1	0	1 (BMP + PRF)	Alendronate (100%)	Osteoporosis (100%)	1.5 mg rhBMP-2	CBCT + gas cromatography	Radiological comparison	18 months
Kim et al.	2022	Case report	3	3	-	Not Reported	Osteoporosis (100%)	1.5 mg rhBMP-2	Panoramic X-Ray + CBCT	Radiological comparison	12 months
Min et al.	2020	Retrospective cohort	26	18	-	Alendronate (42.3%)	Osteoporosis (96.2%)	0.5 mg rhBMP-2	Panoramic X-Ray	Radiological index	12 months
Park et al.	2017	RCT	55	0	30 (BMP+ L-PRF)	Alendronate (54.5%)	Osteoporosis (87.2%)	0.5 mg rhBMP-2	Clinical review + panoramic X-Ray	Own scale	4 months
Jung et al.	2017	Retrospective cohort	17	4	-	Alendronate (Not reported %)	Osteoporosis (100%)	0.5 mg rhBMP-2	Biomarkers + CBCT	Regeneration ratio and corrected regeneration ratio	6 months
Kim et al.	2016	Case report	1	0	1 (BMP + PRF)	Alendronate (100%)	Osteoporosis (100%)	0.5 mg rhBMP-2	Clinical review + panoramic X-Ray	Radiological comparison	3 months
Rahim et al.	2015	Case report	1	1	-	Alendronate (100%)	Osteoporosis (100%)	3.7 mg rhBMP-7	Clinical review + panoramic X-Ray	Radiological comparison	5 years
Cicciù et al.	2012	Case series	20	20	-	Not Reported	Not Reported	6 mg rhBMP-2	Clinical review + panoramic X-Ray	Own scale	1 year

BMP: bone morphogenetic proteins.

### Synthesis of results

#### Patient characteristics

The nine included articles provided a total of 217 patients, of which 101 were treated with rhBMP-2 and one with rhBMP-7. Alendronate was the most used antiresorptive drug, and the most frequent condition for which patients were undergoing treatment was osteoporosis. Table 2 describes the clinical features of patients treated with surgery and BMP versus those who underwent surgery alone.

**Table 2:** Comparison between BMP group and surgery group.

Author	Year	BMP group				Surgery group			
		N patients	Reasson for biphosphonate use	Type of antiresorptive	Treatment outcome	N patients	Reasson for biphosphonate use	Type of antiresorptive	Treatment outcome
Kim et al.	2024	25	Osteoporosis (100%)	Alendronate (36%)	44% complete resolution, 14% delayed, 0% no resolution	39	Osteoporosis	Alendronate (38.5%)	21% complete resolution, 16% delayed, 2% no resolution
Moraes da silva et al.	2022	1	Osteoporosis (100%)	Alendronate (100%)	Complete bone regeneration	0	-	-	-
Kim et al.	2022	3	Osteoporosis (100%)	Oral biphosponates (66.6%)	Complete bone regeneration	0	-	-	-
Min et al.	2020	18	Osteoporosis (96.2%)	Alendronate (33,3%)	Increase radiographic index 11.4±10.6	8	Osteoporosis	Alendronate (75%)	Increase radiographic index 3.27±10.7
Park et al.	2017	30	Osteoporosis (87.2%)	Alendronate (50%)	60% complete resolution, 11% delayed, 1% no resolution	0	-	-	-
Jung et al.	2017	4	Osteoporosis (100%)	Alendronate (100%)	Regeneration ratio 42%	7	Osteoporosis	Alendronate (100%)	Regeneration ratio 17%
Kim et al.	2016	1	Osteoporosis (100%)	Alendronatoe(100%)	Complete bone regeneration	0	-	-	-
Rahim et al.	2015	1	Osteoporosis (100%)	Alendronate (100%)	Complete bone regeneration	0	-	-	-
Cicciù et al.	2012	20	Not reported	Oral and intravenous biphosphonates	Complete bone regeneration	0	-	-	-

BMP: bone morphogenetic proteins.

#### Intervention

Regarding the surgical procedure, all patients underwent thorough curettage to remove bone sequestrum and granulation tissue until new bleeding was observed. Subsequently, absorbable collagen sponges soaked in rhBMP-2, at doses ranging from 0.5 mg/mL to 6 mg/mL (Novosis^®^, CowellBMP^®^, Infuse bone graft^®^), were used as carriers, and the procedure concluded with primary closure of the mucoperiosteal flap. However, slight differences were found among the selected studies. In five studies [16, 25–28], rhBMP-2 was applied as described above, while in two studies [18, 23], patient blood was collected before surgery for the preparation and application of L-PRF, which was ultimately applied over the rhBMP-2-soaked collagen sponges. On the other hand, Rahim et al. used 3.7 mg/mL of BMP-7 [17].

#### Bone regeneration

Bone regeneration after rhBMP application was assessed by comparing post-surgical panoramic radiographs and CBCT with those taken at various follow-up periods ranging from 3.6 to 12 months. Five studies observed bone filling of the defect by comparing radiological tests (panoramic radiographs and CBCT) without employing a specific method to quantify the regeneration [16, 17, 23, 24, 29]. In contrast, two studies used different measurement methods to quantify the amount of new bone formed: the radiographic index and its variation, the regeneration ratio, and the corrected regeneration ratio [26, 27]. The radiographic index was calculated as the ratio of bone density in the defect to the bone density in the adjacent bone. The regeneration ratio was the result of dividing the regeneration area by the initial defect area, and for correction, this value was multiplied by the ratio between the width and height of the defect.


$$\mathrm{Radiografic}\;\mathrm{index}\;(\%)=\frac{\mathrm{radiographic}\;\mathrm{density}\;\mathrm{of}\;\mathrm{the}\;\mathrm{defect}\;\mathrm{site}}{\mathrm{radiographic}\;\mathrm{density}\;\mathrm{of}\;\mathrm{the}\;\mathrm{adjacent}\;\mathrm{site}}\times 100$$

$$\mathrm{Regeneration}\;\mathrm{ratio}\;(\%)=\frac{\mathrm{regenerated}\;\mathrm{area}\;(R)(mm^{2})}{\mathrm{initial}\;\mathrm{defect}\;\mathrm{area}\;(D)(mm^{2})}\times 100$$

$$\mathrm{Corrected}\;\mathrm{regeneration}\;\mathrm{ratio}\;(\mathrm{cor}\;R/D)=\frac{\;\mathrm{regenerated area}\;(R)(mm^{2})}{\mathrm{initial}\;\mathrm{defect}\;\mathrm{area}\;(D)(mm^{2})}\times \mathrm{width}/\mathrm{depth}$$

These authors demonstrated new bone formation, finding statistically significant differences between post-surgical measurements and those taken at 6 months. In the group where rhBMP was applied, Jung et al. observed greater bone formation than in the control group [26]; Min et al. reported an 11.4% variation in the radiographic index in the BMP group compared to 3.27% in the control group [27].

Two studies created a proprietary index to classify outcomes as complete resolution (complete mucosal coverage with no clinical or radiological evidence of MRONJ after 4 weeks post-surgery), delayed resolution (clinical or radiological evidence of MRONJ at 4 weeks but not at 8 weeks), and non-resolution (persistent clinical signs or radiographic progression of MRONJ at 16 weeks). In a study by Park et al., 60% of patients treated with BMP achieved complete resolution, and only one patient did not reach lesion resolution. In the group treated with PRF alone, only 36% of patients achieved complete resolution, and three patients did not achieve lesion healing. In contrast, in another study by Kim et al., 44% of the BMP group achieved complete resolution, 56% showed delayed resolution, and 0% showed non-resolution. This contrasts with the control group, where 58.3% achieved complete resolution, 41% had delayed resolution, and 5.1% showed non-resolution [18, 28].

#### Follow-up

In most studies, both clinical and radiological follow-ups were performed using panoramic radiographs and CBCT, with reviews conducted at 3 or 4 months post-surgery and at 1 year. Among the included articles, the maximum follow-up period was 5 years, as reported by Rahim et al. [17]. During the observation period, lesion resolution was evidenced, with complete mucosal coverage and bone regeneration of the defect post-surgery. Notably, none of the patients treated with rhBMP-2 experienced MRONJ recurrence.

#### Complications

In the selected studies, patients treated with BMPs did not experience complications other than those typically associated with surgical procedures, such as inflammation and bleeding. Only Park et al. reported one patient who did not achieve complete resolution of the lesion.

#### Risk of bias

After evaluating the methodological quality of cohort and case control studies using the NOS [20], it was determined that the studies conducted by Kim, Min and Jung had a low risk of bias (Table 3). The quality analysis of case reports and case series was performed according to the JBI Critical Appraisal Checklist for each type of study, shown in Tables 4 and 5 [21]. Finally, for the RCT by Park et al., the Jadad scale was used, obtaining a score of 2, indicating low methodological quality [22] (Table 6).

**Table 3:** Quality assessment using the Newcastle-Ottawa Scale (NOS) adapted for cohort studies.

Study	Selection				Comparability		Results			Total score
	S1	S2	S3	S4	C1	C2	R1	R2	R3	
Min et al. (2020)	★	★	★	★	★	0	★	★	★	8/10
Jung et al. (2017)	★	★	★	★	★	0	★	★	★	8/10
Kim et al. (2024)	★	★	★	★	★	0	★	★	★	8/10

**Table 4:** Quality assessment using the Joanna Briggs Institute (JBI) adapted for case reports.

	Kim et al. (2022)	Moraes Da Silva et al. (2022)	Kim et al. (2016)	Rahim et al. (2015)
1. Were patient’s demographic characteristics clearly described?	+	+	+	+
2. Was the patient’s history clearly described and presented as a timeline?	+	+	+	+
3. Was the current clinical condition of the patient on presentation clearly described?	+	+	+	+
4. Were diagnostic tests or assessment methods and the results clearly described?	+	+	+	+
5. Was the intervention(s) or treatment procedure(s) clearly described?	+	+	+	+
6. Was the post-intervention clinical condition clearly described?	+	+	+	+
7. Were adverse events (harms) or unanticipated events identified and described?	+	+	-	+
8. Does the case report provide takeaway lessons?	+	+	+	+
Global evaluation	Included	Included	Included	Included

**Table 5:** Quality assessment using the Joanna Briggs Institute (JBI) adapted for case series.

	Cicciù et al. (2012)
1. Were there clear criteria for inclusion in the case series?	-
2. Was the condition measured in a standard, reliable way for all participants included in the case series?	+
3. Were valid methods used for identification of the condition for all participants included in the case series?	+
4. Did the case series have consecutive inclusion for participants?	?
5. Did the case series have complete inclusion of participants?	+
6. Was there clear reporting of the demographics of the participants in the study?	-
7. Was there clear reporting of clinical information of the participants?	-
8. Were the outcomes or follow up results of cases clearly reported?	+
9. Was there clear reporting of the representing site(s)/clinic(s) demographic information?	-
10. Was statistical analysis appropriate?	?
Global evaluation	Included

**Table 6:** Quality assessment using the Jadad Scale for randomized clinical studies.

	Randomization	Blinding	An account of all patients	Total score
Park et al. (2017)	1	0	1	2

## Discussion

Conservative and surgical therapies are the primary treatment approaches for MRONJ. In refractory or recurrent cases, however, extensive bone resections become necessary. Although the literature lacks detailed data on treatment distributions, Sacco et al. conducted a systematic review and meta-analysis involving 36 patients across 28 studies, revealing that 22.2% were stage I, 41.6% stage II, and 19.4% stage III. Among these, 22.2% received conservative treatment alone, 41.6% received conservative management with antibiotics, 30.5% underwent surgical intervention, and 8.8% required resective surgery [3].

Despite the effectiveness of resective procedures, the resulting bone defects are often complex and difficult to reconstruct, especially in patients with impaired bone regeneration. Autogenous bone grafts remain the gold standard for reconstruction but are limited by donor site morbidity and availability, particularly in MRONJ patients [12]. To address these challenges and minimize patient morbidity, alternative regenerative strategies have emerged. These include therapies based on growth factors such as autologous platelet concentrates (APC), teriparatide (TPTD), and notably recombinant human bone morphogenetic protein-2 (rhBMP-2).

Regarding BMPs, they were first introduced in the United States in 2002 after FDA approval for anterior lumbar interbody fusion (ALIF) at a single level. BMP-2 was later approved in 2004 as an alternative to autografts for treating tibial nonunions and in 2007 for maxillofacial reconstructions [30]. The use of rhBMP-2 has shown good results in animal models [31, 32]. Mikai et al. performed extractions on mice treated with zoledronic acid and compared a control group (leaving the socket empty) with the experimental group in which rhBMP-2 was introduced. Immunohistochemical analysis showed that BMP-2 application could accelerate bone formation and reduce alveolar necrosis [33]. Oh et al. conducted a similar study with 20 rats. After 8 weeks of zoledronic acid treatment, extractions were performed, and the animals were divided into two groups: a control group where a collagen sponge was placed in the socket and an experimental group where the sponge was soaked in rhBMP-2. After 8 weeks, micro-CT scans showed greater bone filling and density in the rhBMP-2-treated sockets [34].

Despite the good results in animal models, human studies remain scarce, with the nine articles reviewed in this work being the only ones published to date. All selected articles reported a high success rate in bone regeneration following the use of rhBMP. Most studies conducted clinical and radiological follow-ups at 3–4 months and 1 year post-surgery, with a maximum follow-up of 5 years in one case. During this period, lesion resolution and bone regeneration were observed, with no MRONJ recurrences in patients treated with rhBMP-2.

It is important to observe that the clinical use of BMPs is not universally available, as regulatory approval differs between countries. Moreover, the high cost of recombinant BMPs, poses a major limitation to their widespread clinical use, potentially restricting access to this therapy in standard reconstructive protocols.

Regarding the limitations of this study, the methodological diversity for evaluating bone regeneration in the included studies, ranging from radiological comparison to regeneration index calculations or the development of scales specifically for the study. Consequently, a meta-analysis has not been performed, and the conclusions should be considered with caution. The second limitation is the heterogeneity of the types of publications included, which range from case reports, case series, cohort studies, and case-control studies to a single randomized clinical trial. The final limitation identified is the diversity of BMP applications and doses. In five studies, rhBMP-2 was used alone; in two studies, rhBMP-2 was used in conjunction with the patient’s L-PRF, and in one study, rhBMP-7 was used. The doses also varied among the different articles: three used 1.5 mg/cc, four used 0.5 mg/mL, and one used 6 mg. The study with BMP-7 used a dose of 3.7 mg/mL.

Since the results in humans for rhBMP-2 application for bone regeneration in MRONJ show a favorable trend, more studies with larger sample sizes, uniform methodology, and prolonged follow-up are required to confirm these findings.

## Conclusion

It seems that studies consider rhBMP-2 to be a safe option for bone regeneration in patients with MRONJ. Additional comparative studies are needed, ideally randomized clinical trials with appropriate protocols, adequate sample sizes, and extended follow-up periods, to assess the feasibility of rhbmp-2 in different clinical scenarios.

## Supplementary Material



## Figures and Tables

**Table:** 

	L.H.C.	A.B.G.	C.C.V.	C.M.G.	J.L.Q.	C.M.M.
Conceptualization	x		x			
Data curation	x		x			
Formal analysis	x		x			
Funding acquisition	-	-	-	-	-	-
Investigation	x	x	x			
Methodology	x		x	x		
Project administration			x			x
Resources		x		x		
Software		x		x		
Supervision			x			
Validation					x	x
Visualization				x	x	
Writing – original draft	x		x			
Writing – review and editing			x		x	x
