# Supplementary material for: Use of bmps as a treatment for medication-related maxillary osteonecrosis (mronj): a systematic review
Source: Acta Odontol Scand. 2026 Feb 4;85:45323. doi: 10.2340/aos.v85.45323 (PMC12884370; doi:10.2340/aos.v85.45323)
Supplement: Supplementary file 1 [file AOS-85-45323-s1.pdf]

Supplementary material has been published as submitted. It has not been copyedited or typeset by Acta Odontologica Scandinavica.

Database	Search (July 1st, 2024)
Cochrane Library (Wiley)	**1 Title Abstract Keyword** MeSH descriptor: [BMP] explode all trees<br>**2 Title Abstract Keyword** BMPs OR "bone morphogenetic protein"<br>**3 Title Abstract Keyword** MeSH descriptor: [MRONJ] explode all trees<br>**4 Title Abstract Keyword** "biphosphonate osteonecrosis"<br>**5 Title Abstract Keyword** MeSH descriptor: ["bone regeneration"] explode all trees<br>**6 Title Abstract Keyword** (#1 OR #2) AND (#3 OR #4) AND (#5)
MEDLINE (PubMed)	("bone morphogenetic protein"[Mesh] OR BMP*) AND ("MRONJ" [Mesh] OR "drug related osteonecrosis" OR "biphosponate osteonecrosis") AND ("regeneration" [Mesh] OR "bone regeneration")
Scopus (Elsevier)	TITLE-ABS-KEY ("bone morphogenetic protein" OR BMP*) AND (MRONJ OR "drug related osteonecrosis" OR "biphosponate osteonecrosis") AND ("regeneration" OR "bone regeneration")
Web of Science (Clarivate)	TS ("bone morphogenetic protein" OR BMP*) AND (MRONJ OR "drug related osteonecrosis" OR "biphosponate osteonecrosis") AND ("regeneration" OR "bone regeneration")

Appendix 1. Search strategy adapted for each data base.
